# In-Vitro Antioxidant Potential of Beta-Sitosterol: A Preface

**DOI:** 10.7759/cureus.45617

**Published:** 2023-09-20

**Authors:** Lakshminarayanan Arivarasu

**Affiliations:** 1 Department of Research, Meenakshi Academy of Higher Education and Research, Chennai, IND; 2 Department of Pharmacology, Indira Medical College and Hospitals, Tiruvallur, IND

**Keywords:** in-vitro study, dpph assay, ascorbic acid, antioxidant activity, beta sitosterol

## Abstract

Introduction

Beta-sitosterol, a plant sterol, has been linked to antibacterial and antinociceptive effects. Plant sterols exhibit valuable medicinal properties, including anti-adhesive activities. The dietary composition of sterols includes carbohydrates, lipids, proteins, and various minerals, along with beneficial reinforcements like essential nutrients.

Methodology

This study primarily aims to evaluate the antioxidant potential of beta-sitosterol through assessments using diphenylpicrylhydrazyl (DPPH), hydrogen peroxide (H_2_O_2_), and total antioxidant assays.

Results

The investigation into beta-sitosterol's antioxidant properties revealed a positive correlation between its concentration and antioxidant activity. Similar trends were observed in the present study, indicating an increase in antioxidant activity at lower concentrations.

Discussion and conclusion

Our findings demonstrate that beta-sitosterol exhibits significant antioxidant activity in the tested samples. These results highlight beta-sitosterol's potential as a potent antioxidant and contribute to our understanding of its beneficial effects.

## Introduction

Plant sterols possess unique attributes that enable them to combat microbes, reduce inflammation, and alleviate pain. These qualities stem from their inherent capacity to act as natural antioxidants and influence cellular processes [1]. Recently, these sterols have garnered significant attention due to their remarkable capabilities. Certain sterol variants, such as camposterol and stanols, exhibit notable efficacy in countering cellular threats like pathogens, toxins, genetic mutations, free radicals, and inflammatory and autoimmune responses [2]. These specialized defense mechanisms are crucial for protecting the body against diseases caused by oxidative molecules [3]. Beta-sitosterol is a plant sterol with a structural resemblance to cholesterol. It occurs naturally in various plant sources, including nuts, seeds, and vegetable oils. Often used as a dietary supplement, beta-sitosterol is believed to offer potential health benefits, particularly in cholesterol management. It is thought to reduce the absorption of dietary cholesterol in the intestines, thus potentially lowering levels of LDL ("bad") cholesterol in the bloodstream. Additionally, it finds application in managing benign prostatic hyperplasia (enlarged prostate) and is sometimes considered for its anti-inflammatory properties. However, it's imperative to consult a healthcare professional before using beta-sitosterol supplements to determine their suitability for your specific health requirements and to discuss potential interactions or side effects [4].

The process by which sterols contribute to overall health resembles an intricate molecular interaction that involves the exchange of specific components, ultimately promoting stability within the body. This is particularly vital for maintaining overall well-being and preventing conditions like prostate and breast cancer [5]. Abundantly present in various dietary sources, sterols encompass a diverse nutritional profile comprising carbohydrates, lipids, proteins, and essential minerals. Additionally, they contain essential vitamins such as vitamin C and vitamin E, which confer additional health benefits [6].

Empirical endeavors have been undertaken to assess the efficacy of a specific sterol variant, beta-sitosterol, in combating various biological changes. Study results shows that utilizing methodologies like the DPPH assay, researchers have evaluated its effectiveness in neutralizing detrimental entities, including hydrogen peroxide, a contributor to aging and pathogenesis [7]. Earlier research supports the notion that agents proficient in quelling such harmful changes significantly contribute to preventing degenerative processes. Intriguingly, meticulous examination of beta-sitosterol has unveiled its strong affinity for binding to metals and its propensity to embed within cellular membranes, hinting at potential cytoprotective implications [8].

Given these compelling findings, endeavors to elucidate beta-sitosterol's effectiveness in countering detrimental substances remain a central research objective. Such investigations hold promise for unraveling the extent of beta-sitosterol's ability to mitigate agents that threaten health. Nonetheless, it is crucial to acknowledge that, while these insights are captivating, ongoing scientific inquiry is imperative for fully understanding the intricate mechanisms and potential therapeutic applications of plant sterols across various health contexts.

## Materials and methods

Measurement of total antioxidant activity 

We determined the overall antioxidant capability of beta-sitosterol using a specific method [9]. We prepared 0.3 ml of the sample at different concentrations (ranging from 0.5 to 3 mg/ml). This was mixed with a solution consisting of 3 ml of the regent then placed in a water bath at 95°C for a period of 90 minutes [10]. Subsequently, we assessed the absorbance of all the sample mixtures at 695 nm. The total antioxidant activity was quantified in terms of ascorbic acid equivalents [11].

DPPH assay 

The antioxidant potential of beta-sitosterol was assessed based on its ability to counteract the steady 1,1-diphenyl-2-picryl hydrazyl (DPPH) free radical [12]. We blended the samples with a 2.9 ml solution using varying concentrations (ranging from 0.5 to 3 mg/ml) of diphenylpicrylhydrazyl (DPPH) in methanol (at a concentration of 120 µM). This mixture was then incubated in darkness at 37°C for 30 minutes. The absorbance was measured at 517 nm. The percentage of inhibition of the DPPH free radical (I%) was calculated using the formula: Percentage of Inhibition (I%) = (A blank - A sample) / A blank × 100, where A blank represents the absorbance of the control reaction and A sample is the absorbance of the test compound. These calculations were performed for various concentrations of the sample. As a positive control, we utilized ascorbic acid, and all tests were carried out in triplicate.

Scavenging of hydrogen peroxide (H₂O₂) 

We investigated the ability of beta-sitosterol to counteract hydrogen peroxide using the following method: A solution of 40 mM H₂O₂ was prepared, and its concentration was determined spectrophotometrically. Different concentrations of beta-sitosterol and the standard ascorbic acid were added to 0.6 ml of the 40 mM H₂O₂ solution [13]. After incubating for 10 minutes, the absorbance of H₂O₂ was measured against a blank solution that contained phosphate buffer but lacked hydrogen peroxide.

## Results

The total equivalent antioxidant activity was assessed using ascorbic acid as the reference standard. At a concentration of 25 µg/ml, it displayed a total antioxidant activity (TAA) equivalence of 32%. This equivalence increased to 60% at 50 µg/ml, 85% at 75 µg/ml, 98% at 100 µg/ml, 120% at 125 µg/ml, and 135% at 150 µg/ml. The rise in concentration demonstrated a substantial improvement in total antioxidant activity for the standard ascorbic acid (Table 1).

**Table 1:** The total antioxidant activity equivalent of ascorbic acid (STANDARD) The different concentration levels are depicted along with the percentage of total activity of ascorbic acid. Each value signifies the mean ± SEM. CONC: Concentration AAE: Ascorbic acid equivalent.

CONC in µg/ml	AAE (Standard) % of Inhibition
25 µg/ml	31.87 ±1.27
50 µg/ml	61.42 ±1.30
75 µg/ml	85.37 ±0.81
100 µg/ml	98.29 ±1.22
125 µg/ml	119.57 ±1.31
150 µg/ml	132.86 ±1.28

The effects of beta-sitosterol on a standard substance at different concentration levels. When we used 25 μg/ml of beta-sitosterol, it resulted in inhibition of less than 10%, while the standard substance exhibited 40% inhibition. As we increased the concentration to 50 μg/ml, beta-sitosterol caused a 30% inhibition zone, whereas the standard (ascorbic acid) had a 60% inhibition zone. At a concentration of 75 μg/ml, both ascorbic acid and beta-sitosterol demonstrated inhibitions of 80% and 60%, respectively. Continuing to a concentration of 100 μg/ml, beta-sitosterol achieved 80% inhibition, whereas the standard exhibited 40% inhibition. At the 125 μg/ml concentration, beta-sitosterol displayed approximately 60% inhibition, while the standard showed a higher inhibition of 85%. At the highest concentration of 150μg/ml, beta-sitosterol reached an impressive 90% inhibition, while the standard exhibited a complete 100% inhibition (Table 2).

**Table 2:** The comparative DPPH activity of beta-sitosterol and ascorbic acid (STANDARD) The different concentration levels are depicted with the percentage of inhibition of DPPH activity of beta-sitosterol and ascorbic acid (STANDARD). Each value signifies the mean ± standard error of the mean (SEM).

CONC in µg/ml	Betasitosterol % of inhibition	Standard (ascorbic acid) % of inhibition
25 µg/ml	10.84 ±1.27	37.3 ±1.27
50 µg/ml	31.83 ±1.31	62.7 ±1.31
75 µg/ml	46.42 ±1.28	78.5 ±1.28
100 µg/ml	57.64 ±0.78	83.5 ±0.78
125 µg/ml	71.82 ±1.26	92.4 ±1.26
150 µg/ml	84.29 ±1.24	98.6 ±1.24

The beta-sitosterol's ability to counteract hydrogen peroxide at 25 μg/ml, beta-sitosterol exhibited a significant difference compared to the standard. Progressing to a concentration of 50 μg/ml, beta-sitosterol resulted in a 20% inhibition, while the standard achieved a 50% inhibition. At a concentration of 75 μg/ml, beta-sitosterol demonstrated a 30% inhibition, with the standard exhibiting a higher 65% inhibition. A similar significant difference was observed at the 100 μg/ml concentration level. For the concentration of 125 μg/ml, beta-sitosterol displayed a 45% inhibition, while the standard exhibited a higher 85% inhibition. Finally, at the highest concentration of 150 μg/ml, beta-sitosterol reached a 60% inhibition, and the standard exhibited a complete 100% inhibition (Table 3).

**Table 3:** The comparative H2O2 scavenging activity of beta-sitosterol and ascorbic acid (STANDARD) The different concentration levels are depicted with the percentage of inhibition of H2O2 activity of beta-sitosterol and ascorbic acid (STANDARD). Each value signifies the mean ± standard error of the mean (SEM).

CONC in µg/ml	Beta sitosterol % of inhibition	Standard (ascorbic acid) % of inhibition
25 µg/ml	10.38 ±0.28	43.82 ±1.23
50 µg/ml	23.62 ±0.21	51.09 ±1.31
75 µg/ml	34.58 ±0.32	64.38 ±1.20
100 µg/ml	48.62 ±0.36	72.61 ±1.34
125 µg/ml	57.84 ±0.28	88.53 ±1.28
150 µg/ml	62.49 ±0.24	97.09 ±1.24

## Discussion

Antioxidant research occupies a pivotal role in the scientific exploration of compounds capable of mitigating the detrimental effects of oxidative stress. Ascorbic acid proves to be a versatile asset in in vitro studies, assuming a critical role in maintaining cellular health, exploring oxidative stress and redox reactions, and enriching our understanding of various cellular processes. Its significance spans across multiple domains of research, encompassing cell biology, neuroscience, immunology, and drug development [14]. Oxidative stress arises from an imbalance between reactive oxygen species (ROS) production and the body's intrinsic antioxidant defenses [15]. The potential health implications of oxidative stress, including its association with aging, chronic diseases, and cellular damage, have spurred extensive investigations into natural compounds with antioxidant properties [16].

In our study, the exploration of beta-sitosterol's antioxidant activity aligns seamlessly with the overarching objective of antioxidant research: understanding how naturally occurring compounds can counteract the adverse effects of oxidative stress [17]. The total antioxidant activity findings reveal a concentration-dependent enhancement in the antioxidant activity of ascorbic acid (STANDARD). This suggests that higher concentrations of the compound lead to an increased ability to neutralize oxidative stress (Table 1). The results also highlight that the antioxidant activity of the compound surpasses that of ascorbic acid at certain concentrations [18].

The DPPH inhibition activities of beta-sitosterol and ascorbic acid demonstrate concentration-dependent responses. Higher concentrations of both compounds lead to greater inhibitory activity [19]. However, even at higher concentrations, beta-sitosterol's inhibitory activity falls short of achieving complete inhibition, unlike ascorbic acid. The comparison of DPPH-inhibiting activity between beta-sitosterol and ascorbic acid highlights their distinct antioxidant capacities. Ascorbic acid's well-established role as an antioxidant and its strong DPPH-inhibiting activity contribute to its reputation as a potent free radical scavenger [20]. On the other hand, beta-sitosterol's inhibitory activity, while promising, might not match the efficiency of ascorbic acid in this particular assay (Table 2).

The capability of scavenging of hydrogen peroxide activity, both beta-sitosterol and ascorbic acid, demonstrate H2O2-inhibiting activity, the comparison suggests that ascorbic acid is more efficient in neutralizing the effects of hydrogen peroxide. This can be attributed to its strong antioxidant properties and well-established role as a cellular protector against oxidative stress [21]. Comparing the H2O2 inhibiting activity of beta-sitosterol and ascorbic acid underscores their distinct antioxidant capacities. Ascorbic acid's potent antioxidant and free radical scavenging abilities likely contribute to its superior H2O2 inhibiting effects (Table 3). Beta-sitosterol's inhibition of H2O2 is promising, but it appears to be less potent compared to the well-documented effects of ascorbic acid [22].

Through a comprehensive examination at different concentrations, we have observed distinct impacts of beta-sitosterol in comparison to a standard substance. It is clear that beta-sitosterol possesses a significant ability to inhibit undesirable processes, with its effectiveness varying based on the concentration used. Particularly noteworthy is its strong performance in countering hydrogen peroxide, demonstrating its potential to combat harmful elements. While our primary focus has been on antioxidant activity, we recognize that there is vast unexplored territory in various aspects of its other properties. Moreover, the specific bioactive components responsible for beta-sitosterol's antioxidant effects remain to be identified, necessitating further investigation. The formulation of various applications, such as gels or mouthwashes, holds promise for unlocking the full potential of beta-sitosterol's capabilities. Our study underscored a significant distinction in the antioxidant effects of beta-sitosterol when compared to the standard. Furthermore, we are yet to identify the specific bioactive site in the molecular structure of beta-sitosterol that could be accountable for its potentially beneficial effects [23]. Further research may encompass the formulation of different applications, such as gels or mouthwashes, with the potential to conduct clinical trials to assess their potential benefits [24].

## Conclusions

Our exploration of the effects of beta-sitosterol has provided us with compelling insights; proceeding to further study is the logical next step to reveal its practical benefits and implications. As we conclude this phase of our study, we acknowledge that beta-sitosterol's journey from the laboratory to practical applications is just beginning, offering numerous opportunities to deepen our understanding and enhance human well-being.
